# Early phase (day 1–day 3) profile and clinical significance of circulating miR-101 and miR-146a in adult sepsis

**DOI:** 10.1590/1806-9282.20252209

**Published:** 2026-07-31

**Authors:** Özlem Aldemir, Hatun Öztürk Çerik, Burcu Bayyurt, Murtaza Öz

**Affiliations:** 1 Sivas Numune Hospital, Department of Infectious Diseases and Clinical Microbiology – Sivas, Türkiye.; 2 Ordu University Training and Research Hospital, Department of Infectious Diseases and Clinical Microbiology – Ordu, Türkiye.; 3 Sivas Cumhuriyet University, Department of Medical Biology – Sivas, Türkiye.; 4 Sivas Cumhuriyet University, Department of Infectious Diseases and Clinical Microbiology – Sivas, Türkiye.

**Keywords:** Sepsis, MicroRNAs, Inflammation, Biomarkers, Gene expression, Immunity, innate

## Abstract

**OBJECTIVE:**

The aim of this study was to investigate serum miR-101 and miR-146a expression levels in adult sepsis, compare them with healthy controls, and assess their relationships with inflammatory markers and disease severity.

**METHODS:**

In total, 15 adults meeting Sepsis-3 criteria and 15 healthy controls were prospectively enrolled. Serum miR-101 and miR-146a levels were quantified by quantitative polymerase chain reaction within 24 h using the 2^-^∆^Ct^ method and reassessed on day 3. Associations with clinical, laboratory, and disease severity parameters were analyzed.

**RESULTS:**

On day 1, miR-101 expression was significantly higher in the sepsis group than in controls (1.17 vs. 0.14; p=0.011). miR-146a levels were lower in sepsis but did not differ significantly (p=0.152). Neither miRNA showed a significant change from day 1 to day 3 (miR-101: p=0.776; miR-146a: p=0.191). miR-101 demonstrated positive correlations with procalcitonin, white blood cell count, C-reactive protein, creatinine, and a negative correlation with albumin. Levels were markedly higher in patients with cardiovascular comorbidities (ρ=0.622; p=0.013). miR-101 correlated positively with Acute Physiology and Chronic Health Evaluation II (ρ=0.412; p=0.024), while no association was observed with Sequential Organ Failure Assessment. miR-146a showed no meaningful correlations with clinical or laboratory parameters.

**CONCLUSION:**

miR-101 is significantly elevated in adult sepsis and reflects systemic inflammatory burden and disease severity; however, given its limited specificity, it should be considered a complementary biomarker rather than a standalone diagnostic or prognostic tool. Its stable expression between days 1 and 3 suggests a sustained early phase molecular response. miR-146a shows no significant change and has limited diagnostic relevance in adult sepsis. Larger multicenter studies are needed to validate these findings.

## INTRODUCTION

Sepsis is a life-threatening condition characterized by a dysregulated host response to infection, leading to organ dysfunction and high mortality^1^. Its global burden and its position as the leading cause of mortality in intensive care units highlight the critical need for early diagnosis and timely, targeted treatment^2^. The condition is marked by widespread endothelial activation, an uncontrolled inflammatory response, and disruption of microvascular integrity, ultimately resulting in multiorgan dysfunction^3,4^. Consequently, there remains a pressing need for rapid, reliable, and biologically informative diagnostic markers^5^.

MicroRNAs (miRNAs) are short non-coding RNA molecules that regulate gene expression at the post-transcriptional level and play important roles in multiple steps of the immune response. Among these, miR-101 and miR-146a have emerged as key regulatory molecules. miR-101 has been associated with pro-inflammatory processes, although evidence regarding its diagnostic or prognostic relevance in adult sepsis remains limited. miR-146a, a crucial negative regulator of innate immunity, participates in feedback control of inflammation; however, clinical studies have yielded inconsistent findings across different patient populations^6,7,8,9,10,11,12,13,14,15,16,17,18^.

Circulating miRNAs are recognized as dynamic regulators that may change over time and reflect the biological course of disease^19^. In rapidly progressive conditions such as sepsis, monitoring miRNA kinetics may provide insights into the stage of the inflammatory response and early treatment effects. Therefore, miR-101 and miR-146a levels were measured on day 1 and day 3 to evaluate early phase (first 72 h) expression patterns in adult sepsis.

To our knowledge, no prior study has examined miR-101 and miR-146a expression in sepsis within the Turkish population, underscoring the originality of this work. This study aimed to assess the diagnostic and potential prognostic value of miR-101 and miR-146a in adult sepsis, compare their expression levels with healthy controls, examine their associations with clinical and laboratory parameters, and evaluate their early phase (days 1–3) temporal changes.

## METHODS

### Participants

This prospective observational study was conducted between June 2024 and December 2024 in the Infectious Diseases and Clinical Microbiology units of Sivas Numune Hospital and Ordu University Faculty of Medicine. In total, 15 adult patients (≥18 years) who met the Sepsis-3 diagnostic criteria, including an increase of ≥2 points in the Sequential Organ Failure Assessment (SOFA) score, were enrolled^1^. A priori power analysis (α=0.05, β=0.10) indicated that 15 individuals per group provided adequate statistical power.

Exclusion criteria included acute myocardial infarction, pulmonary embolism, malignancy, trauma, HIV infection, immunosuppressive therapy within the past 6 months, pregnancy or breastfeeding, and death within the first 24 h of admission.

Healthy volunteers aged ≥18 years with no comorbidities and normal physical examinations. Written informed consent was obtained from all participants.

### Data collection and definitions

Demographic characteristics (age, sex, BMI), smoking history, and comorbidities were recorded. Admission laboratory parameters included serum creatinine, albumin, white blood cell count (WBC), C-reactive protein (CRP), procalcitonin (PCT), platelet count (PLT), and lactate. Blood culture results obtained within the first 24 h were documented. Acute Physiology and Chronic Health Evaluation II (APACHE II) and SOFA scores were assessed at admission and at 72 h.

### Sampling and serum preparation

Blood samples from sepsis patients were collected within 24 h of admission and again at 72 h; controls were sampled at enrollment. Samples were centrifuged at 1,600 g for 10 min at 4°C, and the supernatants were clarified by a second centrifugation at 12,000 g for 10 min. Serum aliquots were stored at -80°C until analysis.

### Serum miRNA isolation

Total RNA was extracted from serum using a commercial isolation kit in accordance with the manufacturer’s protocol. RNA concentration and purity were assessed by measuring the A260/A280 ratio. Samples were stored at -20°C until further processing.

### Complementary DNA synthesis and quantitative polymerase chain reaction analysis

RNA samples were diluted to 100 ng/μL and subjected to genomic DNA elimination followed by reverse transcription. Quantitative polymerase chain reaction (qPCR) was performed using SYBR Green chemistry with specific primers for miR-101, miR-146a, and the reference gene RNU6. Reactions were conducted according to the manufacturer’s instructions. RNU6 served as the endogenous control. RNU6 was chosen as the internal reference due to its frequent use in serum/circulating miRNA qPCR workflows and its compatibility with the primer set and assay conditions applied in this study. We acknowledge, however, that reference selection remains challenging in circulating miRNA analyses. Expression levels were calculated using the 2^-^∆^Ct^ method; the target miRNA expression level was determined as 2^-[Ct(serum miRNA)-Ct(U6)]^.

### Follow-up and clinical outcomes

All patients were followed for 28 days and classified as “survivors” or “non-survivors” based on their outcome at the end of the follow-up period.

### Statistical analysis

Statistical analyses were performed using IBM SPSS Statistics version 23.0 (IBM Corp., Armonk, NY, USA). The distribution of continuous variables was assessed using the Kolmogorov-Smirnov and Shapiro-Wilk tests. Normally distributed variables were compared using the independent samples t-test, while non-normally distributed variables were analyzed with the Mann-Whitney U test. Paired measurements were evaluated with the Wilcoxon signed-rank test. Categorical variables were compared using the chi-square or Fisher’s exact test. Correlations were assessed using Spearman’s rank correlation coefficient (ρ). A p<0.05 was considered statistically significant.

The study was approved by the Clinical Research Ethics Committee of Sivas Cumhuriyet University (Approval No: 2024-03/16; Date: March 26, 2024).

## RESULTS

A total of 15 sepsis patients and 15 healthy controls were included in the study. The groups were similar in age, sex, BMI, and smoking status, while comorbidities were significantly more frequent in the sepsis group (p<0.001). APACHE II scores were markedly higher in sepsis patients (18.6±4.9 vs. 6.4±2.1; p<0.001), and the median day 1 SOFA score was 3 (2–3). Laboratory analyses showed significantly higher WBC, PCT, CRP, creatinine, and lactate levels, and lower albumin levels in the sepsis group (all p<0.01). Baseline demographic, clinical, and laboratory characteristics of the study groups are summarized in Table 1. Regarding microRNA expression, day 1 miR-101 levels were significantly elevated in sepsis patients compared with controls [1.17 (0.21–4.11) vs. 0.14 (0.00–0.43); p=0.011], whereas miR-146a levels were lower but not significantly different (p=0.152) (Table 2). On day 3, miR-101 and miR-146a levels were 1.39 (0.20–3.29) and 0.52 (0.15–0.97), respectively, with no significant temporal change from day 1 (p=0.776 and p=0.191). Correlation analyses demonstrated that miR-101 levels were positively associated with cardiovascular comorbidity (ρ=0.622; p=0.013), PCT (ρ=0.509; p=0.004), WBC (ρ=0.455; p=0.011), CRP (ρ=0.500; p=0.005), and creatinine (ρ=0.436; p=0.016), and negatively associated with albumin (ρ=-0.466; p=0.009). miR-101 correlated positively with APACHE II (ρ=0.412; p=0.024) but showed no association with SOFA (p=0.659) (Table 3). miR-146a demonstrated no significant correlations with clinical or laboratory parameters. Survival analysis could not be performed due to only one death, and comparison of miRNA levels by blood culture pathogens was not feasible because of the limited and uneven distribution of isolates.

**Table 1:** Baseline demographic, clinical, and laboratory characteristics of the study population.

Variable	Sepsis group (n=15	Healthy control group (n=15)	p-value
Demographic and clinical characteristics			
Age (years), mean±SD	69±12.8	65±6.4	0.291
Male sex, n (%)	5 (33.3)	5 (33.3)	1.000
Presence of comorbidity, n (%)	13 (86.6)	0 (0)	**<0.001**
BMI >25 kg/m^2^, n (%)	10 (66.6)	10 (66.6)	1.000
Current smoking, n (%)	2 (13.3)	3 (20.0)	1.000
SOFA score, day 1, median (IQR)	3 (2–3)	—	—
APACHE II score, day 1, mean±SD	18.6±4.9	6.4±2.1	**<0.001**
Laboratory parameters			
WBC (10^3^/μL), median (IQR)	11.9 (8.5–18.5)	7.0 (5.8–7.7)	**<0.001**
PLT (10^3^/μL), mean±SD	215±123	258±59	0.271
PCT (ng/mL), median (IQR)	9.9 (3.0–36.0)	0.04 (0.02–0.05)	**<0.001**
CRP (mg/L), mean±SD	259±81	2.1±1.4	**<0.001**
Serum creatinine (mg/dL), mean±SD	1.81±0.62	0.75±0.13	**<0.001**
Albumin (g/L), mean±SD	30±4.3	42±2.8	**<0.001**
Lactate (mmol/L), mean±SD	2.2±0.9	1.3±0.5	**0.006**

Data are presented as mean±standard deviation or median (interquartile range), as appropriate. BMI: body mass index; SOFA: Sequential Organ Failure Assessment; APACHE II: Acute Physiology and Chronic Health Evaluation II; WBC: White blood cell count; PLT: platelet count; PCT: procalcitonin; CRP: C-reactive protein; SD: standard deviation; IQR: interquartile range. A p<0.05 was considered statistically significant. Bold values indicate statistical significance (p<0.05).

**Table 2:** miR-101 and miR-146a expression levels (day 1 and day 3).

Variable	Sepsis group (median/IQR or mean±SD)	Healthy control group (median/IQR)	p-value
miR-101 (day 1)	1.17 (0.21–4.11)	0.14 (0.00–0.43)	**0.011**
miR-146a (day 1)	0.67 (0.41–1.07)	1.32 (0.42–8.28)	0.152
miR-101 (day 3)	1.39 (0.20–3.29)	—	—
miR-146a (day 3)	0.52 (0.15–0.97)	—	—
miR-101 (day 1 vs. day 3, sepsis)	Day 1: 1.17 (0.21–4.11) • Day 3: 1.39 (0.20–3.29)	—	0.776
miR-146a (day 1 vs. day 3, sepsis)	Day 1: 0.67 (0.41–1.07) • Day 3: 0.52 (0.15–0.97)	—	0.191

Data are presented as median (IQR) for non-normally distributed variables. Between-group comparisons were performed using the Mann-Whitney U test, and within-group temporal changes were evaluated using the Wilcoxon signed-rank test. No day 3 measurements were obtained for the healthy control group. SD: standard deviation; IQR: interquartile range. Bold values indicate statistical significance (p<0.05).

**Table 3:** Correlation between miR-101 expression levels and clinical/laboratory parameters.

Parameter	Spearman’s ρ	p-value
Comorbidities (presence)		
Diabetes mellitus presence	-0.155	0.582
Hypertension presence	-0.349	0.202
Cardiovascular disease presence	**0.622**	**0.013**
Chronic obstructive pulmonary disease presence	-0.186	0.508
Chronic kidney disease presence	-0.182	0.517
Neurological disease presence	0.247	0.374
Clinical severity scores		
SOFA score	-0.124	0.659
APACHE II score	**0.412**	**0.024**
BMI >25 kg/m^2^	0.082	0.667
Laboratory parameters		
PCT (ng/mL)	**0.509**	**0.004**
WBC (10^3^/μL)	**0.455**	**0.011**
CRP (mg/L)	**0.500**	**0.005**
Platelets (10^3^/μL)	-0.194	0.300
Serum creatinine (mg/dL)	**0.436**	**0.016**
Albumin (g/L)	**-0.466**	**0.009**
Lactate (mmol/L)	0.334	0.071

APACHE II: Acute Physiology and Chronic Health Evaluation II; BMI: body mass index; CRP: C-reactive protein; PCT: procalcitonin; PLT: platelet count; SOFA: Sequential Organ Failure Assessment; WBC: white blood cell count. Spearman’s rank correlation coefficient (ρ) was used. Comorbidities were analyzed as binary variables (presence/absence). A p<0.05 was considered statistically significant. Bold values indicate statistical significance (p<0.05).

## DISCUSSION

In this study, the expression profiles and early kinetics of miR-101 and miR-146a were evaluated in adult sepsis, and the findings demonstrated a significant elevation of miR-101 during sepsis. This increase was strongly associated with inflammatory burden and clinical severity parameters. The markedly higher miR-101 levels observed in patients with cardiovascular comorbidities suggest that this molecule may reflect not only systemic inflammatory stress but also cardiac strain. The higher miR-101 levels in individuals with cardiovascular disease and its stable expression between days 1 and 3 indicate a sustained early phase molecular response. The absence of studies assessing serial miR-101 measurements in adult sepsis underscores the originality of the present findings.

The increase in miR-101 observed in our study aligns with both experimental and clinical data. In sepsis models, miR-101-3p has been shown to rise in parallel with IL-1β, IL-6, and TNF-α and to correlate with inflammatory load^6^. Similar elevations have been reported in neonatal and pediatric sepsis, supporting the view that miR-101 represents a conserved inflammatory signature across age groups^20^. However, the rise in miR-101 is not specific to sepsis; markedly higher levels have been described in conditions characterized by intense cytokine activation, such as adult-onset Still’s disease^7^. This pattern suggests that miR-101 reflects global inflammatory stress rather than a disease-specific mechanism. Experimental work showing that miR-101 inhibition regulates inflammation via DUSP1-mediated suppression of the MAPK p38, and NF-κB pathways further support the notion that miR-101 participates in both the amplification and regulation of inflammatory signaling^6^.

The positive correlations between miR-101 and PCT, CRP, WBC, and creatinine, along with its negative correlation with albumin, indicate that this molecule integrates signals of immune activation, metabolic stress, and organ dysfunction. The positive association with APACHE II—contrasting with the lack of correlation with SOFA—suggests that miR-101 may reflect overall disease severity rather than acute organ failure. Taken together, miR-101 appears to be a meaningful molecular indicator of inflammation and clinical severity in adult sepsis, although its limited specificity restricts its utility as a standalone biomarker. Accordingly, miR-101 may serve more effectively as a complementary molecular indicator reflecting the biological burden and clinical severity of sepsis rather than as an independent diagnostic marker.

In contrast, miR-146a did not demonstrate a significant change in adult sepsis. Considering the heterogeneity of findings in the literature, this result is noteworthy. In pediatric populations, miR-146a suppression has been consistently reported^17,18,21^, suggesting that developmental differences in immune regulation may substantially influence its expression. Adult studies, however, show divergent results, with some reporting increases and others showing no meaningful change^9,11,15,22^. The neutral findings of Puskarich et al.^22^ align with the present study.

Several biological and methodological factors may underlie this heterogeneity. First, given that miR-146a acts as a negative regulator of the TLR4/NF-κB axis, its expression may respond more robustly in Gram-negative infections^9,23^. The heterogeneous pathogen distribution in our cohort may therefore have diluted the measurable biological signal. Second, common comorbidities in adults (such as diabetes, hypertension, and cardiovascular disease) may influence baseline miRNA levels, masking sepsis-specific changes. Third, the timing of sampling may not fully capture the early kinetic fluctuations of miR-146a, as experimental data suggest that expression changes occur rapidly within the first hours after LPS stimulation^24^.

One of the distinguishing strengths of this study is the use of serial measurements for both miR-101 and miR-146a. Temporal miRNA analyses in adult sepsis are scarce; thus, day 1 and day 3 assessments provide insight into early phase expression stability. The absence of an early phase (days 1–3) change in miR-146a further suggests that this molecule may exhibit a stable early response in adult sepsis, distinct from the patterns observed in pediatric populations.

The study’s limitations include the small sample size, low mortality rate, and heterogeneous distribution of infectious agents, which may have particularly affected the statistical power for miR-146a analyses. Additionally, comorbid conditions that may alter baseline miRNA profiles must be considered. These limitations highlight the need for larger, multicenter studies with improved temporal resolution to delineate the true clinical value of these miRNAs. In addition, although RNU6 was selected as the endogenous control in line with common practice, RNU6 may exhibit variability in circulating miRNA measurements; therefore, our relative quantification findings should be interpreted within this context. Due to resource/budget constraints, parallel normalization/validation using multiple endogenous reference controls could not be implemented in the same cohort; future studies are encouraged to validate our findings using these approaches.

## CONCLUSION

This study presents the first clinical dataset from Türkiye evaluating the expression profiles of miR-101 and miR-146a in adult sepsis. miR-101 was significantly elevated during sepsis, and its increase was associated with inflammatory burden, cardiovascular comorbidities, and APACHE II score. The notably higher miR-101 levels observed in patients with cardiovascular disease suggest that this molecule may reflect not only systemic inflammation but also cardiac stress. Furthermore, the early phase stability of miR-101 between day 1 and day 3 supports its potential as a sustained indicator of inflammatory activity during the early phase of sepsis.

In contrast, the lack of significant change in miR-146a expression indicates that the suppressed response reported in pediatric populations may not extend to adult phenotypes, suggesting that miR-146a acts as a context-dependent immune modulator in adults.

The limited diagnostic accuracy of both miRNAs when considered individually reflects the biological heterogeneity of sepsis. Therefore, integrating miR-101 and miR-146a into multimarker panels (alongside inflammatory and clinical parameters) may provide stronger diagnostic and prognostic value.

## Data Availability

The datasets generated and/or analyzed during the current study are available from the corresponding author upon reasonable request.
